# Characterization of Pre-Breeding Wheat (*Triticum aestivum* L.) Germplasm for Stripe Rust Resistance Using Field Phenotyping and Genotyping

**DOI:** 10.3390/plants12183239

**Published:** 2023-09-12

**Authors:** Basharat Ul Islam, Saba Mir, Mohammad Saleem Dar, Gazala H. Khan, Asif B. Shikari, Najeeb ul Rehman Sofi, Fayaz Mohiddin, Mohammad Ashraf Ahangar, Intikhab Aalum Jehangir, Satish Kumar, Gyanendra Singh, Shabir H. Wani

**Affiliations:** 1 Division of Genetics and Plant Breeding, Faculty of Agriculture, Wadura, Sopore, Sher-e-Kashmir University of Agricultural Sciences and Technology, Srinagar 190025, Jammu and Kashmir, India; basharatulislamagsc@gmail.com (B.U.I.); asifshikari@gmail.com (A.B.S.); 2 Mountain Research Centre for Field Crops, Genetics and Plant Breeding, Sher-e-Kashmir University of Agricultural Sciences and Technology, Srinagar 190025, Jammu and Kashmir, India; mirsaba90@gmail.com (S.M.); saleemdar0001@gmail.com (M.S.D.); gazalakhan5818@gmail.com (G.H.K.); najeeb_sofi@rediffmail.com (N.u.R.S.); famohiddin@rediffmail.com (F.M.); mashrafjs@gmail.com (M.A.A.); intikhabaalum@gmail.com (I.A.J.); 3 ICAR-Indian Institute of Wheat and Barley Research, Karnal 132001, India; gysingh@gmail.com

**Keywords:** wheat, stripe rust, disease resistance breeding, *Yr* genes, molecular markers

## Abstract

Wheat is highly affected by stripe rust disease, particularly under cooler environments, and the losses can reach up to 100 percent depending on the intensity of infection and the susceptibility of the genotype. The most effective method to manage this disease is the use of resistant varieties. In the present study, 192 wheat genotypes were evaluated for stripe rust resistance under field conditions and also in a laboratory using molecular markers. These lines included pre-breeding germplasm developed for rust resistance and some high-yielding commercially grown wheat varieties. Out of 192 genotypes, 53 were found to be resistant, and 29 showed moderate resistance reaction under field conditions, whereas the remaining genotypes were all either moderately susceptible or susceptible. Under controlled conditions, out of 109 genotypes, only 12 were found to be resistant to all the six virulent/pathogenic pathotypes. Additionally, a selection of 97 genotypes were found resistant in field screening and were subjected to molecular validation using the markers linked to major R-genes, *viz*., *Yr5*, *Yr10*, *Yr15* and *Yr17*. Nine genotypes possessed the *Yr5* gene, twelve had the *Yr10* gene, fourteen had the *Yr15* gene and thirty-two had the *Yr17* gene. The resistance genes studied in the current study are effective in conferring resistance against stripe rust disease. The genotypes identified as resistant under both field and controlled conditions can be used as sources in stripe rust resistance breeding programs.

## 1. Introduction

One of the world’s most important and widely grown food crops is bread wheat (*Triticum aestivum* L.) [1,2]. A significant portion of the global population relies on wheat for their daily nutrition and food [3,4]. As per the third advance estimate of production of major crops for agricultural year 2022–2023 released by the Ministry of Agriculture and Farmers Welfare, Government of India wheat production is estimated to touch a record of 112.74 million tonnes [5]. Three important wheat-growing states in India are Uttar Pradesh, Punjab and Haryana, which contribute about 80 percent of the country’s total wheat production [6]. Presently, global climatic changes have become a threat to wheat yield and grain quality as many abiotic factors and disease pressure have become constraints [7,8,9]. Major diseases of wheat are the fungal pathogens, mostly including prevalent stripe rust, powdery mildew, Karnal bunt, etc. [2,10]. Among the fungal diseases, wheat rusts, threaten wheat production in the wheat-dominating regions of northern India. Depending on the kind of rust, environmental factors, the growth stage of the crop at which the disease arises, and the level of host resistance, rusts can reduce wheat production by up to 100% [11]. Among the rusts, stripe rust is a devastating foliar disease grown in subtropical and cooler temperate regions of the world, and it often breaks into epidemics in such ecologies [12,13,14]. Wheat stripe rust disease is caused by the pathogen *Puccinia striiformis* W. var. *striiformis*, which is further differentiated based on their host specialization known as *tritici* Eriksson [15]. The vulnerability of the wheat variety, the start of the epidemic, the severity of rust, the length of time rust persists on the wheat, and the temperature during grain filling all affect the damage caused by stripe rust [16,17]. Cultivation of susceptible cultivars along with early infection of disease can cause 100 percent yield loss [18]. Limited grain output is the result of the pathogen’s ability to induce foliar damage, reduced foliage, chlorophyll degradation, and wrinkled grains [19]. It occurs regularly in a severe form on susceptible varieties in the Trans-Gangetic Plains (covering Haryana, Western Uttar Pradesh, Punjab and Delhi) and the Western Himalayan Region (covering Himachal Pradesh, Punjab, Jammu and Kashmir and Ladakh). Due to the global climate change scenario, yellow rust disease epidemics in the Western Himalayas are occurring more frequently than before. The evolution of new races of *Puccini striiformis* f. sp. *tritici* in the Western Himalayan region is responsible for the bust of many popular, high-yielding varieties grown in the region, including HS 240, Shalimar Wheat-1, Shalimar wheat-2, PBW-343 and many other cultivars [20]. Further, as many of the cultivars lack resistance to new pathotypes and since many of the ruling varieties share a common ancestry, which makes them more vulnerable to serious disease outbreaks. So far, 86 (Yr1-Yr86) permanently designated resistance genes, 100 temporarily designated genes, and 363 quantitative trait loci (QTLs) have been reported for stripe rust resistance in wheat [21,22]. Out of 84 permanently designated resistance genes only 19 R genes viz., Sr60, Yr15/YrG303/YrH52, Lr1, Lr10, Lr21, Lr22a, Sr13, Sr21, Sr22, Sr33, Sr35, Sr45, Sr46, SrTA1662, Sr50, Yr5/YrSP, Yr7, Yr10 and YrAS2388R, controlling all-stage resistance to different wheat fungal pathogens have been cloned. Of these resistant genes, *Yr5*, *Yr10*, *Yr15* and *Yr17* are highly effective to prevalent pathotypes [22]. Additionally, the tagged markers for these genes are available and can be used to identify the presence or absence of these genes in the germplasm (http://wheat.pw.usda.gov/, accessed on 29 July 2023). The all-stage resistance (ASR) genes *Yr5*, *Yr10*, and *Yr15* are strong and effective options for utilization in resistance breeding, as these have been effective across the globe for many years now. Hexaploid *Triticum aestivum* ssp. spelta var *album* (TSA) is where the stripe rust resistance gene *Yr5* was first identified. It is a race-specific R-gene that works at all plant growth stages and is located on chromosome 2BL. It was first identified on chromosome 2B and later localized to the long arm of that chromosome, 21 cM from the centromere [23]. The dominant stripe rust resistance gene Yr10, which is situated on chromosome 1BS, is 2 cM away from the Rg1 locus, which imparts brown glume color, and 5 cM away from the Gli-1B locus [23]. It was first identified in the wheat lines PI 178383 and Moro. Yr10 resistance gene still effectively confers resistance to stripe rust in many places of the world. All races have been found to be affected by this gene in China, India, Pakistan, Iran, the United States, and Kazakhstan [23]. *Triticum dicoccoides* accession G-25 harbored the dominant stripe rust resistance gene *Yr15*, positioned on chromosome 1BS. *Yr15* provides wide-ranging resistance towards more than 3000 genetically distinct *Pst* isolates around the globe [24]. The 2NS/2AS or *Lr*37/*Yr*17/*Sr*38 translocation is another popular introgression that carries the adult plant resistance (APR) gene *Yr17*. It is still effective against some prevalent pathotypes of Pst. in Indian and also provides resistance to other rusts and biotic pathogens. Though there are a large number of genes available, their utilization is restricted, because most of these genes are dominant, race-specific and, therefore, do not provide durable resistance independently. Therefore, the identification of novel sources of resistance in a cultivar is of foremost importance for effective disease control. Moreover, the availability of molecular markers linked to genes and their utilization in tracking genes is a strategic approach in rust resistance breeding. 

One of the effective approaches to exploit genetic resistance against the disease would be to identify and utilize novel sources of resistance for controlling stripe rust disease of wheat in Jammu and Kashmir and Ladakh regions of the Western Himalayas. The occurrence of deadly stripe rust in this region can have a long impact on the breadbasket of India (North Western Plains), as the disease can spread to these areas. Thus, screening and use of resistance germplasm is a prime objective of breeding programs. Keeping in view the above fact, a set of wheat germplasm lines was screened under hotspot conditions of Kashmir in order to identify the lines carrying resistance under open field conditions and under artificial inoculation conditions in a greenhouse. The lines were also validated using molecular markers linked to known loci that confer resistance against stripe rust.

## 2. Results

### 2.1. Evaluation of Stripe Rust Resistance

The analysis of variance (Table 1) suggested a significant impact of block and treatments (years) on the study at *p*-value < 0.001. Response to stripe rust infection substantially varied across the genotypes due to the significant effect of genetic factors. The tested genotypes were also found to differ significantly from the tested check lines.

### 2.2. Field Evaluation

**Figure 1:**
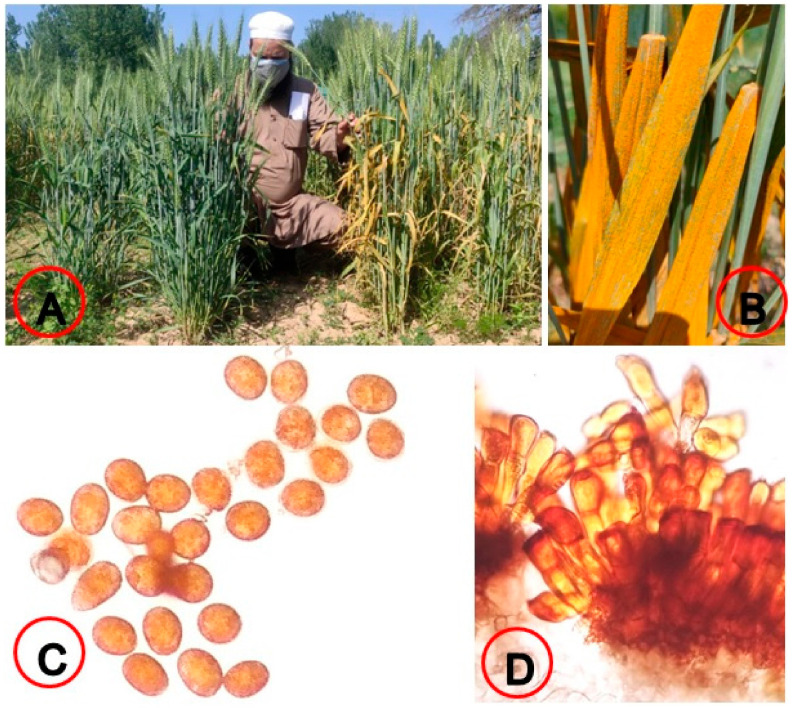
(**A**) Resistant vs. susceptible genotypes under field conditions; (**B**) yellow rust spores on leaf; (**C**) yellow rust Urideospores; (**D**) yellow rust Teliospores.

The symptoms of stripe (yellow) rust disease were first observed on the leaves of Agra-local, Shalimar Wheat 1and PBW-343, which were used as susceptibility checks (Figure 1). The estimated final rust intensity (FRI) and AUDPC values for the few resistant wheat genotypes are summarized in Table 2. The high intensity of yellow rust was revealed during the seasons of 2020–2021 and 2021–2022, as denoted by the high and low values of FRI and AuDPC for wheat genotypes, respectively. The reactions of the wheat genotypes towards yellow rust were significantly diverse. During the 2020–2021 season, the FRI values ranged from resistant (5R), the lowest limit, to susceptible (90S), the highest limit, and during 2021–2022 cropping season, from immune (0I), the lowest limit, to moderately susceptible (40MS), the highest limit (Table S1).

The minimum and maximum AUDPC values for resistant wheat genotypes were in the range of 1 to 250 (Table S1). Corresponding to this, the range of the lowest and highest AUDPC limits for moderately resistant wheat genotypes ranged between 251 and 500. For the moderately susceptible (MS) and susceptible (S) genotypes, the minimum and maximum limits of AUDPC were determined to be between 501–750 and more than 751, respectively. During 2020–2021 and 2021–2022, based on the final rust intensity and AUDPC scores, it was revealed that 53 genotypes showed resistance to yellow rust disease, 29 genotypes were moderately resistant, 54 genotypes were moderately susceptible, and 56 genotypes were susceptible (Table S1)

The field observations indicated that the disease symptoms first appeared in the susceptibility checks (viz. Agra-local, Shalimar Wheat-1 and PBW-343). Final rust intensity (FRI) and AUPDC values indicated the availability of the highly resistant and susceptible genotypes in our germplasm collection (Table S1). Furthermore, FRI and AUPDC scores were lesser during 2020–2021 in contrast to 2021–2022 (Table 2). At least 26 genotypes showed an FRI score of 5R in the growing season 2020–2021; however, 15 of these showed immunity (0I) towards yellow rust in the succeeding growing season. These 15 genotypes showed zero values for AUPDC due to the fact that they were immune. Finally, in the present study, 29.1%, 28.1%, 15.1%, and 27.6% of the genotypes were found to be susceptible, moderately susceptible, moderately resistant, and resistant (Table S1).

### 2.3. Greenhouse Evaluation

Further, a subset of one hundred and nine (109) wheat genotypes comprising resistant and moderately resistant genotypes based on the field evaluation was used for greenhouse evaluation. These genotypes were screened in controlled environments against the six most virulent and prevalent races of yellow rust pathogen (*P. striiformis*). All of the 109 test genotypes showed varied reactions against these six pathotypes *viz*., 7S0, 23S119, 110S119, 110S84, 47S119(T), and 46S119 of yellow rust under greenhouse-controlled epiphytotic conditions (Table S2). It was observed that 104 genotypes were resistant towards the pathotype 7S0 race, 17 genotypes were resistant against 23S119, 21 genotypes against 110S119, 54 genotypes towards 110S84, and 62 genotypes towards 47S119, whereas 43 genotypes were resistant to pathotype 46S119. Furthermore, 12 genotypes *viz*., KWB-8, KWB-43, KWB-64, KWB-91, KWB-95, KWB115, KWB-126, KWB-129, KWB-132, KWB-138, KWB-140, and KWB-147 were resistant to all six pathotypes (Table 3). Further, these 12 genotypes were also found to be resistant to yellow rust under field screening conditions.

### 2.4. Marker Based Analysis 

**Figure 2:**
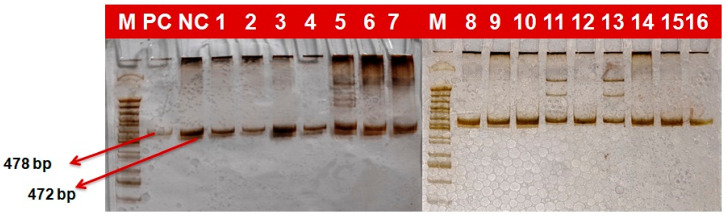
PCR-amplified products of markers STS7/8 detecting Yr5 gene in wheat genotypes (478 bp size fragment as resistant and 472 bp fragment as susceptible).

**Figure 3:**
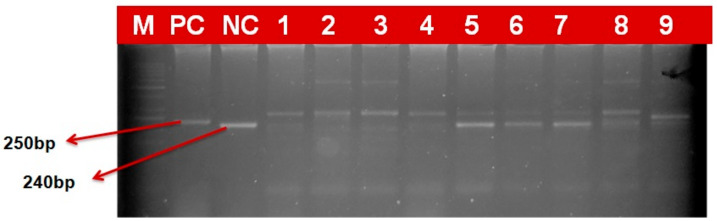
PCR-amplified products of markers XPSP3000 detecting Yr10 gene in wheat genotypes (260 bp size fragment as resistant and 240 bp fragment as susceptible).

**Figure 4:**
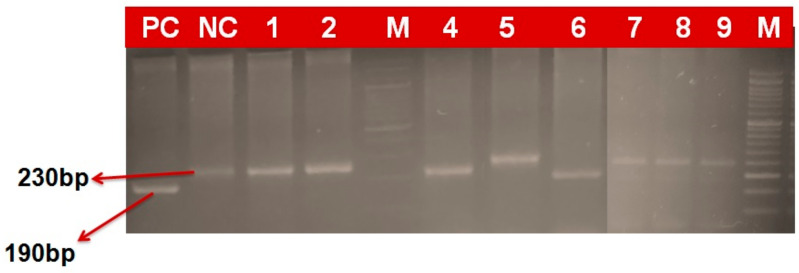
PCR-amplified products of markers Xbarc8 detecting Yr15 gene in wheat genotypes (190 bp size fragment as resistant and 230 bp fragment as susceptible).

**Figure 5:**
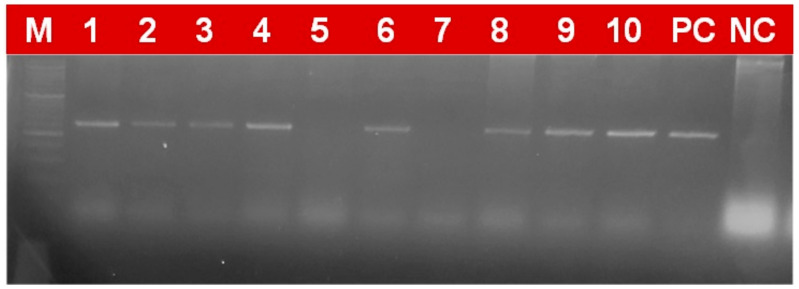
PCR-amplified products of markers VENTRUIP/LN2 detecting Yr17 gene in wheat genotypes (presence and absence for resistant and susceptible, respectively. M: 50 bp DNA Size standard (Fermentas, LA, USA).

In the current study, 97 wheat genotypes identified based on field screening as resistant and moderately resistant, along with some susceptibility check lines, were screened with molecular markers linked with four yellow rust resistance genes. Gel electrophoresis revealed differential and comparable banding patterns, which were used to score the genotypes for the presence/absence of these genes (Figure 2, Figure 3, Figure 4 and Figure 5). The results obtained after the molecular marker screening for effective yellow rust genes *viz*., *Yr5*, *Yr10*, *Yr15* and *Yr17* are presented in Table S3. For *Yr5*, the SSR marker (*STS7/8*) amplified two types of bands, which were observed at 478 bp (resistant) and 472 bp (susceptible). The *Yr5* gene was present in nine (9.3%) genotypes, which showed a single band of 478 bp size. Microsatellite marker *Xpsp3000* located 1.3 cM proximal to *Yr10* was used to resolve the existence or absence of the *Yr10* gene. *Xpsp3000* produced two types of bands, 260 bp for resistant genotypes, while 240 bp was for susceptible. During screening, the *Yr10* gene was found to be present in 12 genotypes (12.4%), with an amplified band of 260 bp, while 85 (87.6%) genotypes did not carry the gene, as they amplified a fragment of 240 bp size, which is for susceptibility. In the case of the *Xbarc8* marker for the *Yr15* gene, two alleles with the size of 190 and 230 bp were amplified. The expected fragment size for the *Xbarc8* locus linked to the resistant allele of the *Yr15* gene was found to be 190 bp. Among the genotypes, the expected PCR product (190 bp) was amplified in 14 genotypes (14.4%). The fragment sized 230 bp was non-specific and amplified in the remaining 83 (85.6%) genotypes. Using the *VENTRIUP/LN2*, the presence of the *Yr17* gene was investigated. A total of 32 (38.9%) genotypes were found to possess the *Yr17* gene, as evidenced by the presence of a 262 bp resistant band. As *VENTRIUP/LN2* is a dominant marker, the remaining 65 (67.05%) genotypes did not yield any band, and as a result, they were confirmed to not carry the *Yr17* gene.

The wheat genotypes *viz*., KWB-8, KWB-43, KWB-64, KWB-91, KWB-95, KWB-115, KWB-126, KWB-129, KWB-132, KWB-138, KWB-140, and KWB-147 showed the presence of three *Yr* genes. Genotypes KWB-8, KWB-64, KWB-91, KWB-115, KWB-126, and KWB-138 possessed *Yr10*, *Yr15* and *Yr17*; genotypes KWB-43, KWB-95, and KWB-132 possessed *Yr5*, *Yr10* and *Yr17* genes; genotypes KWB-140, and KWB-147 had *Yr5*, *Yr10* and *Yr15* genes; and genotype KWB-129 carried *Yr5*, *Yr15* and *Yr17* genes. Similarly, the wheat genotypes *viz*., KWB-40, KWB-137, and Unnat PBW-703 showed the presence of two *Yr* genes, with genotype KWB-40 carrying *Yr10* and *Yr17* and genotype KWB-137 carrying *Yr5* and *Yr15,* while Unnat PBW-703 carried *Yr10* and *Yr15* genes (Table S3).

Twelve promising genotypes *viz*., KWB-8, KWB-43, KWB-64, KWB-91, KWB-95, KWB-115, KWB-126, KWB-129, KWB-132, KWB-138, KWB-140, and KWB-147, possessed three effective *Yr* genes each and were identified as resistant under field and controlled conditions against the six most virulent races (Table 4).

## 3. Discussion

Many biotic stresses hamper wheat production; among them, wheat rusts are notorious for their deadly and pervasive nature. In the context of India, stripe rust (caused by *Puccinia striiformis* f. sp. *tritici*) is a menace, particularly in Northern India, where wheat covers an area of more than 10 million ha [25]. Extensive screening of indigenous and exotic wheat germplasm under natural field and epiphytotic controlled conditions has resulted in the identification of novel sources of rust resistance in wheat, including stripe rust resistance [26,27]. The identification of effective donors for wheat rust resistance is required for the mapping of novel alleles that can be utilized in marker-assisted selection to develop rust-resistant varieties of wheat. Although 86 genes for resistance to rust have been characterized, the location effectiveness of the genes may vary so that not all the genes can be utilized freely. The phenotyping process is also important for understanding the racial profile or general virulence behaviour of the local isolates.

In the present investigation, wheat genotypes exhibited a wide range of responses to the yellow rust pathogen, ranging from extremely susceptible to highly resistant, which adhered to the results of earlier investigations [28,29]. Agra-local, Shalimar Wheat-1, and PBW-343 (susceptible hosts), utilized as disease spreaders, were the first to show rust symptoms. The incidence of yellow rust was noted during both the years 2020–2021 and 2021–2022, with greater intensity evidenced during the first year. The possibility of identifying genotypes harboring resistance to virulent races of yellow rust has already been explored by many researchers [30,31]. These studies assume that field-based screening for resistance is essential for the development of novel cultivars with long-lasting resistance. Presently, measures such as disease intensity, FRI, and AUDPC are utilized to quantify the yellow rust disease and its progression over time. The genotypes were divided into four groups based on the values of FRI and rust intensity. A total of 53 (27.6%) genotypes displayed resistance to the yellow rust disease; 29 (15.1%) genotypes were moderately resistant, 54 (28.1%) genotypes were moderately susceptible, and 56 (29.2%) genotypes were susceptible. The data also revealed that in both cropping seasons (2020–2021 and 2021–2022), disease intensity, FRI, and AUDPC were highest in Agra-Local followed by PBW-343, which were used as disease spreaders.

Based on the field screening of 192 wheat genotypes for yellow rust resistance in two consecutive cropping seasons (2020–2021, and 2021–2022), resistant, moderately resistant, and some susceptible lines, were again tested under greenhouse conditions against the six most virulent races of yellow rust pathogen in the Indian subcontinent *viz*., 7S0, 238S119, 110S119, 110S84, 47S119 (T), and 46S119. It was found that only 12 wheat genotypes *viz*., KWB-8, KWB-43, KWB-64, KWB-91, KWB-95, KWB-115, KWB-126, KWB-132, KWB-138, KWB-140, and KWB-147 were resistant against all these six races of *P. striiformis* f. sp. *tritici.* Similarly, Li et al. [32] tested 115 wheat genotypes in the field and under controlled conditions against three virulent races of yellow rust (CYR32, CYR33, and V26) found in China. They found that 53 (46.1%) cultivars (lines) were resistant to all three races at all stages, and 16 (13.9%) cultivars (lines) were resistant at adult plant stage towards stripe rust. Additionally, a total of 135 genotypes of wheat were examined for yellow rust during the growing season of 2008/2009 under field conditions by Tabassum [33], and it was found that 25 lines had the lowest FRI (percent) and AUDPC (percent < 260). When Mateen et al. [34] investigated 150 lines under field conditions for stripe rust, they discovered that 64 lines and varieties were resistant, 42 lines displayed resistance, and the other lines were susceptible to yellow rust. In another investigation, Hassan et al. [35] evaluated 22 commercial wheat varieties against leaf rust under field conditions, and only one variety was found to be resistant.

Phyto-pathological approaches based on symptomology are not always successful in identifying resistance genes. The environment has a significant impact on field evaluation. A varied and reliable presence of resistant genes must serve as the cornerstone when developing wheat cultivars resistant to rust. Molecular markers linked to disease resistance will be the most effective way to pinpoint the elements that contribute to disease resistance. Due to availability and low-cost, the high-throughput molecular marker platforms, marker-assisted selection (MAS), is a practical method for identifying resistance genes. The wheat genotypes that demonstrated resistance and moderate resistance against the yellow rust disease under naturally infected field circumstances were chosen for molecular characterization. To ensure the accuracy of the data, certain susceptible lines and resistant genotypes were also chosen for this experiment. Hence, a subset of the present genotypes comprising 97 germplasm lines was validated for the presence of four effective yellow rust resistance genes *viz*., *Yr5*, *Yr10*, *Yr15*, and *Yr17* by using gene-linked markers *STS7/8*, *XPSP3000*, *Xbarc8*, and *VENTRUIP/LN-2*, respectively. These genes have been found to confer resistant phenotypes in wheat cultivars. In the current study, we observed three types (single-, two-, and three-gene) combinations among the germplasm under study. The stripe rust resistance gene *Yr5* was derived from *Triticum spelta var album* and is located on chromosome 2BL. It is a race-specifc R-gene effective at both seedling and adult plant growth stages [23]. The gene *Yr5* was found in nine genotypes, indicating the presence of a resistant gene. Our results showed a similar trend with the results reported by Chen et al. [36] and Zhou et al. [37]. STS-9/10, developed by Chen et al. [36], co-segregates with the Yr5 locus and amplified fragments of 439 or 433 bp for resistant or susceptible plants, respectively. In India, to date, the *Yr5* gene is effective against all prevalent races and can be an effective source for stripe rust resistance when used singly or in combination with other resistant genes [22]. As *Yr5* is a race-specific seedling resistance gene, it should be used in combination with other effective genes and/or with race-non-specific adult-plant resistance genes. Such a combination could provide durable resistance. The dominant stripe rust resistance gene *Yr10*, which is situated on chromosome 1BS, is 2 cM away from the Rg1 locus, which imparts brown glume color, and 5 cM away from the Gli-1B locus [23]. Microsatellite marker *Xpsp3000*, located 1.3 cM proximal to *Yr10*, was used to indicate the presence or absence of the *Yr10* gene in wheat genotypes [38]. The co-dominant microsatellite marker *XPSP3000* has been reported to be used for identifying individual genotypes at any growth stage [39] for *Yr10* marker-aided selection [40]. The resistance gene *Yr10* was originally found in wheat lines PI178383 and Moro. In the present study, twelve genotypes were found to have *Yr10*, while the remaining genotypes did not carry a resistance gene. In similar research findings, 34 genotypes were found to carry the *Yr10* gene when using *XPSP3000* markers with the desired band size (260 bp) [41]. The *Yr10* gene was also found to be effective against all the Pst races prevalent in India, Iran, China, Pakistan, and the United States [42]. *Triticum dicoccoides* accession G-25 was found to contain the dominant stripe rust resistance gene *Yr15*, which was located on chromosome 1BS [23]. Yr15 gives broad-spectrum resistance against more than 3000 genetically varied Pst isolates from all over the world, including contemporary races like ‘Warrior’ (race DK09/11), which is currently endangering wheat output [24]. Further, Murphy et al. [43] discovered *Xbarc8* as being a linked marker to the *Yr15* gene. *Xbarc8* is a key molecular tool for determining the presence of the *Yr15* gene in almost all backgrounds examined [43]. In the current study, among the 97 genotypes, only 14 genotypes carried the *Yr15* gene, as per the desired product size amplified. The presence of the *Yr17* gene in wheat genotypes was investigated using the *VENTRIUP/LN2* marker. This gene was first discovered on the 2NS chromosome of *Triticum ventricosum* T, and it was first introduced into the “VPM1” variety of winter bread wheat, involving the 2NS/2AS translocation [44,45]. The *Yr17* gene has been found to be linked to *Lr37* and *Sr38* genes, which confer resistance to leaf and stem rusts, respectively. As a result, wheat lines and cultivars expressing the *Yr17* gene exhibit resistance to rusts [46]. The *Yr17* gene was found to be present in 32 genotypes out of the 97 genotypes (Supplementary Table S3). In *VENTRUIP/LN2*, a dominant marker linked to *Yr17*, a 262 bp fragment was amplified in resistant genotypes, whereas susceptible genotypes lacked this amplicon. Similar results concerning the amplification of a 262 bp fragment in resistant genotypes were recently observed in HD2967, Trinkria; 6-11, HD2967 + LrTrk NILs, while this fragment was not amplified in Agra Local and Kharchia Local [47]. Pinpointing commendatory alleles for stripe rust resistance is a criterion for augmenting the resistance of present-day wheat varieties via introgression and garnering many favorable alleles from the wheat gene pool by using molecular markers. In the present study, few genotypes harbored multiple favorable stripe rust resistance genes, which will serve as sources for the deployment of durable stripe rust resistance in locally adapted, high-yielding but susceptible cultivars.

## 4. Materials and Methods

### 4.1. Plant Materials and Experimental Design 

The experimental materials used for the present study comprised 192 wheat genotypes, including six and three resistant and susceptible checks, respectively. The details of the genotypes used for the present study are given in Table S4. The field experiments were conducted at MRCFC Khudwani (33°70′ N, longitude 75°10′ E) under Sher-e-Kashmir University of Agricultural Sciences and Technology of Kashmir (Jammu and Kashmir, India), situated at an altitude of 1590 m amsl. Wheat genotypes were evaluated using an Augmented Block Design (ABD) during two consecutive growing seasons, 2020–2021 and 2021–2022. The field plots consisted of 1.5 m long rows (six rows planted at a spacing of 0.20 cm). All the recommended agronomic practices were followed to raise the crop. 

### 4.2. Field Evaluation of Yellow Rust Resistance

The experimental site, MRCFC, SKUAST-K, Khudwani, is a well-known yellow rust epidemic area in the Union territory of Jammu and Kashmir region of Northern Hill Zone of India, where the most predominant and virulent yellow rust pathotypes have been reported, and very high levels of disease incidence are recorded every year [48]. To ensure a uniform yellow rust inoculum spread, susceptible checks, like Agra-local, Shalimar Wheat 1, and PBW-343, were planted after every 8 plots of the test genotypes, and at the periphery of the experimental field, infector rows were grown. Furthermore, the test genotypes were dusted with rust spores by shaking the infected plants over them at fortnightly intervals [49]. The yellow-rust-infected plants used were collected from different trials at MRCFC Khudwani, and before every dusting, the crop field was adequately sprayed with water.

The modified Cobb scale [50] was used to determine the intensity of yellow rust, which is scored from 0% to 100% to determine the percentage of leaf tissue covered in yellow rust, and the intensity was recorded. Yellow rust disease intensity data were recorded after every fifteen days from the first appearance of symptoms on the leaves of the susceptibility checks (Agra-local, Shalimar Wheat-1, and PBW-343). Since intensity is determined by comparing the visual modified scale scores with disease intensity of the test genotypes, the readings taken are based on average estimation. Disease intensity and response readings were recorded as follows:

1. 0% = Immune.
2. 5 to 10% of intensity = resistant.
3. 10 to 29% of intensity= moderately resistant.
4. 30 to 59% of intensity = moderately susceptible.
5. 60% or more of intensity= susceptible.

The yellow rust intensity was recorded on 15 randomly selected plants per plot of a wheat genotype. The total yellow rust intensity per genotype was then calculated by using the following formulae

Disease intensity (%) = [sum (n × v)]/[(N) × (G)] × 100

where n = frequency, in particular the score class of the modified Cobb scale; V = score of the rating class of the modified Cobb scale; N = total number of plants randomly taken per genotype; and G = maximal disease score class on modified Cobb scale.

#### Calculation of AuDPC

The area under the disease progress curve was determined by using the following formula developed by CIMMYT.

$$AUDPC=\overset{n-1}{\underset{i=1}{\sum }}\left\lfloor \frac{\left(\mathrm{xi}\right)+\left(\mathrm{xi}+1\right)}{2}\right\rfloor \left(t_{i+1}-t_{i}\right)$$

where Xi denotes disease intensity on the date i; ti describes time in days between ith and date i + 1st; and n indicates the total number of times that disease was recorded.

### 4.3. Seedling Stage Evaluation of Yellow Rust under Epiphytotic Conditions in the Greenhouse

Under greenhouse conditions, seedling stage stripe rust evaluation was carried out for a subset of 109 wheat genotypes at the Regional Station, ICAR- Indian Institute of Wheat & Barley Research (IIWBR), Flowerdale, Shimla, India. The seedlings were grown in aluminum bread pans using a mixture of sterilized satisfactory loam and farmyard manure (3:1). A susceptible genotype was planted after each 20 wheat genotypes. For every wheat line, 5 seeds were sown in three replications. Proper susceptible checks for stripe rust and differentials to affirm the purity of the pathotypes were additionally maintained. The seedlings of wheat genotypes were grown in spore-proof greenhouses at a 20 ± 2 °C temperature, with 60–70% relative humidity and 12 h of daylight. Seven-day-old seedlings that had two primary leaves were gently rubbed parallel to the leaf veins with fingers prior to inoculation. The seedlings were inoculated with 100 mg urediospores of individual races suspended in 10 mL light-grade mineral oil (Soltrol 170) (Chevron Phillips Chemicals Asia Pvt. Ltd., Singapore) by using a glass atomizer or sprayer. The oil was then allowed to evaporate for 30 min, and the inoculated seedlings were subsequently housed for two days in mist chambers with a temperature of 17 ± 2 °C and a relative humidity of more than 90%. The seedlings were then moved into a greenhouse and incubated at a temperature of 17 ± 2 °C with a relative humidity greater than 75% [51]. Following the modified Stakman scale method [52], responses of the wheat genotypes to yellow rust disease were noted 14 days after inoculation. Infection types having small hypersensitive flecks to small uredial pustules were recorded as being highly resistant infection types; 1–2 (small hypersensitive flecks to small-moderate uredial pustules with chlorosis) were considered resistant; and infection types of 33+ to 4+ (moderate to large uredial pustules without chlorosis) were considered susceptible (Table S5). Infection type 33+ indicated where both 3 and 3+ pustules were found together.

### 4.4. Molecular Characterization of Presence/Absence Polymorphism in Yr5, Yr10, Yr15 and Yr17

The genomic DNA was extracted from the fresh leaves of individual plants at the two-leaf seedling stage using the CTAB method [53]. The quantity of DNA was checked by loading 5 µL of stock DNA mixed with 3 µL of loading buffer into separate wells on 0.8% agarose gel. The PCR reaction mix (10 μL) used was composed of a DNA template, forward and reverse primers, ThermoScientific DreamTaq PCR Master Mix (2X) (Waltham, MA, USA) and nuclease-free water (sterile water). PCR was performed using the following thermal regimes: denaturation at 94 °C for 5 min, 35 cycles (annealing for 30 s; annealing is unique to a primer since different primers bind to template DNA at different temperatures), and an extension step at 72 °C. The PCR products were loaded on 2.5% agarose gel or an 8% polyacrylamide gel. The germplasm lines were validated at *Yr* loci with the help of linked markers, as described in Table 5.

### 4.5. Statistical Analyses

Analysis of variance was carried out with the SAS program (SAS Institute, Cary, NC, USA) for the test variables *viz*., and disease intensity and area under disease progress curve (AUDPC) were performed as per procedure. The statistical model consists of the following mathematical expression.

Yij = μ + gi + rj + gij + eij(1)

where:

Yij = observation of ith individual (g = 1 − i) in jth replication (r = 1 − k);
μ = general mean;
gi = effect of the ith genotype;
rj = effect of jth replication;
eij = random error related with ijth observations.

## 5. Conclusions

From the above findings, it has become apparent that the values of AUDPC and disease intensity are highest for highly susceptible genotypes and lowest for resistant genotypes. Yellow rust resistance (*Yr*) genes studied in the current research project are effective in conferring resistance against yellow rust disease. The lines possessing effective yellow rust resistance (*Yr*) genes shall be utilized for gene deployment strategies. The twelve promising wheat genotypes *viz*., KWB-8, KWB-43, KWB-64, KWB-91, KWB-95, KWB-115, KWB-126, KWB-129, KWB-132, KWB-138, KWB-140, and KWB-147, possessed three effective *Yr* genes each and were identified as being resistant under field and controlled conditions against the six most prevalent and virulent races. These and similar earlier findings will be useful for plant pathologists’ and plant breeders’ endeavors in the Northern Hill Zone of India and neighboring regions to advancing the inclusive resistance to stripe rust by delivering novel resistant sources and culminate in the value of using field screening, artificial inoculation and markers to improve selection accurateness in national wheat breeding programs.

## Figures and Tables

**Table 1:** Mean sum of squares of disease intensity and area under the disease progress curve (AuDPC) for growing seasons 2020–2021 and 2021–2022.

Source of Variation	d.f.	Disease Severity	AUDPC
Blocks (eliminating treatments)	6	21.97 ***	12,732.45 **
Treatments (ignoring blocks)	191	909.80 ***	218,729.73 **
Treatments (eliminating blocks)	191	851.05 ***	203,064.18 **
Checks	5	6728.99 ***	1,620,622.46 **
Varieties	185	690.75 ***	163,947.62 **
Check vs. variety (C vs. V)	1	12,337.633 **	3,343,956.32 *
**Critical difference @ 5% level of significance Comparison Type**		**Dis. Intensity**	**AuDPC**
Between two controls mean		1.05	38.98
Between two varieties in same block		2.80	103.11
Between two varieties not in same block		3.01	111.37
Between variety and control		2.30	84.19

If a *p*-value is less than 0.05, it is flagged with one star (*). If a *p*-value is less than 0.01, it is flagged with 2 stars (**). If a *p*-value is less than 0.001, it is flagged with three stars (***).

**Table 2:** Final rust intensity and AUDPC scores of the wheat genotypes.

Genotype	FRI (2020–2021)	AuDPC (2020–2021)	FRI (2021–2022)	AuDPC (2021–2022)	Reaction Type
KWB-7	5R	42.0	5R	54.0	R
KWB-8	5R	35	0I	0.0	R
KWB-28	5R	42.0	5R	49.0	R
KWB-29	5R	28.0	5R	49.0	R
KWB-43	5R	6.0	0I	0.0	R
KWB-54	5R	84.0	5R	35.0	R
KWB-87	5R	49.0	5R	28.0	R
KWB-91	5R	35	0I	0.0	R
KWB-95	5R	77.0	0I	0.0	R
KWB-112	5R	30.8	5R	18.0	R
KWB-113	5R	30.8	5R	28.0	R
KWB-114	5R	30.8	5R	30.8	R
KWB-115	5R	77.0	0I	0.0	R
KWB-118	5R	30.5	5R	14.0	R
KWB-126	5R	32.0	0I	0.0	R
KWB-129	5R	35.0	0I	0.0	R
KWB-132	5R	35.0	0I	0.0	R
KWB-137	5R	48.7	5R	21.0	R
KWB-138	5R	60.2	0I	0.0	R
KWB-140	5R	77.0	0I	0.0	R
KWB-143	5R	35.0	5R	21.0	R
KWB-147	5R	35.0	0I	0.0	R
Avocet-Yr5	5R	21.0	0I	0.0	R
Avocet-Yr10	5R	21.0	0I	0.0	R
Avocet-Yr15	5R	21.0	0I	0.0	R
DWB-187	5R	42.0	0I	0.0	R

Abbreviations: I = immune; R = resistant.

**Table 3:** Disease reactions of the most promising wheat genotypes against the six most virulent and aggressive races of *P. striiformis*.

Genotype	7S0	238S119	110S119	110S84	47S119 (T)	46S119
KWB-8	R	R	R	R	R	R
KWB-43	R	R	R	R	R	R
KWB-64	R	R	R	R	R	R
KWB-91	R	R	R	R	R	R
KWB-95	R	R	R	R	R	R
KWB-115	R	R	R	R	R	R
KWB-126	R	R	R	R	R	R
KWB-129	R	R	R	R	R	R
KWB-132	R	R	R	R	R	R
KWB-138	R	R	R	R	R	R
KWB-140	R	R	R	R	R	R
KWB-147	R	R	R	R	R	R

R = resistant

**Table 4:** Most promising wheat genotypes obtained after thorough screenings.

Sl. No	Genotype	*Yr* Gene/s Present	Greenhouse Screening	Field Screening
1	KWB-8	*Yr10*, *Yr15*, *Yr17*	Resistant against all six races	Resistant
2	KWB-43	*Yr10*, *Yr5*, *Yr17*	Resistant against all six races	Resistant
3	KWB-64	*Yr10*, *Yr15*, *Yr17*	Resistant against all six races	Resistant
4	KWB-91	*Yr10*, *Yr15*, *Yr17*	Resistant against all six races	Resistant
5	KWB-95	*Yr10*, *Yr5*, *Yr17*	Resistant against all six races	Resistant
6	KWB-115	*Yr10*, *Yr15*, *Yr17*	Resistant against all six races	Resistant
7	KWB-126	*Yr10*, *Yr15*, *Yr17*	Resistant against all six races	Resistant
8	KWB-129	*Yr5*, *Yr15*, *Yr17*	Resistant against all six races	Resistant
9	KWB-132	*Yr10*, *Yr5*, *Yr17*	Resistant against all six races	Resistant
10	KWB-138	*Yr10*, *Yr15*, *Yr17*	Resistant against all six races	Resistant
11	KWB-140	*Yr5*, *Yr10*, *Yr15*	Resistant against all six races	Resistant
12	KWB-147	*Yr5*, *Yr10*, *Yr15*	Resistant against all six races	Resistant

**Table 5:** The genes for yellow rust resistance and the linked markers along with the primer sequences used for the validation of wheat germplasm.

Type of Marker	Annealing Temperature	Primer Sequence R (5′ to 3′)	Primer Sequence F (5′ to 3′)	Primer Name	Chr. No	Gene	Ref.
STS	45 °C	GCAAGTTTTCTCCCTAT	GTACAATTCACCTAGAT	STS-7/8	2B-L	*Yr5*	[32]
SSR	52 °C	GATATAGTGGCAGCAGGATACG	GCAGACCTGTGTCATTGGTC	XPSP3000	1B-S	*Yr10*	[54]
SSR	52 °C	GCGGGGGCGAAACATACACATAAAAACA	GTACAATTCACCTAGAGT	X-barc8	1B-S	*Yr15*	[38]
SSR	65 °C	TGCAGCTACAGCAGTATGTACACAAAA	AGGGGCTACTGACCAAGGCT	VENTRIUP, LN2	2A-S	*Yr17*	[40]

## Data Availability

The data is contained within the manuscript and Supplementary Materials.

## Supplementary Materials

The following supporting information can be downloaded at: https://www.mdpi.com/article/10.3390/plants12183239/s1, Table S1: Disease reaction type and Phytopathological symptoms observations; Table S2: Details of wheat genotypes used for the present study; Table S3: final rust intensity (FRI) and AUDPC scores of the wheat genotypes; Table S4: Disease reactions of the wheat genotypes against the six most virulent and aggressive races of *P. striiformis*; Table S5: Molecular screening of wheat genotypes for presence of four effective yellow rust resistance genes.
