# Supplementary material for: Characterization of Pre-Breeding Wheat (Triticum aestivum L.) Germplasm for Stripe Rust Resistance Using Field Phenotyping and Genotyping
Source: Plants (Basel). 2023 Sep 12;12(18):3239. doi: 10.3390/plants12183239 (PMC10538134; doi:10.3390/plants12183239)
Supplement: Supplementary file 1 [file plants-12-03239-s001.zip › plants-2528372-supplementary.pdf]

plants

MDPI

Supplementary Materials

Table S1. Final rust intensity (FRI) and AUDPC scores of the wheat genotypes.

Genotype	FRI (%) (2020-21)	AuDPC (2020-21)	FRI (%) (2021-22)	AuDPC (2021-22)	Reaction Type
KWB-1	40MS	616.0	10R	147.0	MS
KWB-2	20MR	235.2	5R	49.0	MR
KWB-3	50MS	661.5	20R	234.5	MS
KWB-4	30MR	425.2	10R	105.0	MR
KWB-5	20MR	227.5	5R	63.0	MR
KWB-6	40MS	570.5	20R	241.5	MS
KWB-7	5R	42.0	5R	54.0	R
KWB-8	5R	35.0	0I	0.0	R
KWB-9	70S	997.5	40MS	518.0	S
KWB-10	50MS	745.5	30MR	409.5	MS
KWB-11	50MS	631.7	10MR	350.0	MS
KWB-12	40MS	516.2	10R	225.7	MS
KWB-13	50MS	724.5	10R	241.5	MS
KWB-14	60MS	738.5	20MR	255.5	MS
KWB-15	40MR	546.0	10R	169.7	MR
KWB-16	50MS	749.0	30MR	357.0	MS
KWB-17	40MS	533.7	10R	154.0	MS
KWB-18	10R	231.0	5R	35.0	R
KWB-19	50MS	703.5	10R	213.5	MS
KWB-20	30MR	378.0	10R	112.0	MR
KWB-21	50MS	717.5	10R	234.5	MS
KWB-22	40MS	502.2	10R	84.0	MS
KWB-23	50MS	742.0	30MR	423.5	MS
KWB-24	50MS	749.0	30MR	437.5	MS
KWB-25	30MR	486.5	10R	91.0	MR
KWB-26	40MS	584.5	10R	224.0	MS
KWB-27	20MR	255.0	5R	42.0	MR
KWB-28	5R	42.0	5R	49.0	R
KWB-29	5R	28.0	5R	49.0	R
KWB-30	10R	105.0	5R	49.0	R
KWB-31	60MS	738.5	30MR	357.0	MS
KWB-32	40MS	665.0	10R	155.7	MS
KWB-33	10R	84.0	5R	21.0	R
KWB-34	40MS	647.5	10R	176.7	MS
KWB-35	20MR	251.0	10R	98.0	MR
KWB-36	50MS	743.7	30MR	435.7	MS
KWB-37	30MR	435.7	10R	150.5	MR
KWB-38	10R	112.0	5R	21.0	R
KWB-39	10R	140.0	5R	35.0	R
KWB-40	10R	126.0	5R	42.0	R
KWB-41	40MS	542.5	10R	169.7	MS
KWB-42	60S	812.0	20MR	322.0	S
KWB-43	5R	6.0	0I	0.0	R
KWB-44	40MS	773.5	10R	197.7	MS
KWB-45	50MS	749.0	20R	269.5	MS
KWB-46	30MR	644.0	10R	112.0	MR
KWB-47	70S	1071.0	30MR	364.0	S
KWB-48	50MS	1113.0	20R	241.5	MS
KWB-49	70S	1102.5	20MR	444.5	MS
KWB-50	60S	913.5	20MR	402.5	MS
KWB-51	70S	1142.4	30MS	514.5	MS
KWB-52	40MS	619.5	10R	190.7	MS
KWB-53	20MR	371.0	10R	91.0	MR

KWB-54	5R	84.0	5R	35.0	R
KWB-55	50MS	714.0	20MR	241.5	MS
KWB-56	50MS	742.0	20MR	266.0	MS
KWB-57	30MR	448.0	10R	126.0	MR
KWB-58	10R	248.5	5R	84.0	R
KWB-59	10R	248.5	5R	84.0	R
KWB-60	60MS	745.5	20MR	375.9	MS
KWB-61	10R	140.0	5R	35.0	R
KWB-62	40MS	556.5	10R	176.7	MS
KWB-63	40MS	609.0	10R	152.9	MS
KWB-64	10R	133.0	5R	35.0	R
KWB-65	10R	140.0	5R	47.0	R
KWB-66	70S	1039.5	30MS	509.2	S
KWB-67	70S	1036.0	30MS	511.0	S
KWB-68	70S	1064.0	30MS	539.0	S
KWB-69	5R	77.0	5R	21.0	R
KWB-70	10R	126.0	5R	35.0	R
KWB-71	70S	1083.2	30MS	558.4	S
KWB-72	5R	7.0	5R	21.0	R
KWB-73	10R	147.0	5R	21.0	R
KWB-74	10R	91.0	5R	35.0	R
KWB-75	30MR	418.2	10R	147.7	MR
KWB-76	10R	98.0	5R	7.0	R
KWB-77	5R	14.0	5R	28.0	R
KWB-78	5R	14.0	5R	28.0	R
KWB-79	10R	122.5	5R	28.0	R
KWB-80	10R	147.0	5R	21.0	R
KWB-81	20MR	391.3	10R	84.0	MR
KWB-82	20MR	385.0	10R	84.0	MR
KWB-83	70S	1078.0	30MS	511.0	S
KWB-84	70S	1172.5	30MS	571.2	S
KWB-85	20R	260.5	10R	122.5	MR
KWB-86	10R	142.0	5R	35.0	R
KWB-87	5R	49.0	5R	28.0	R
KWB-88	5R	35.0	5R	21.0	R
KWB-89	5R	35.0	5R	20.0	R
KWB-90	70S	1172.5	30MS	567.0	S
KWB-91	5R	35.0	0I	0.0	R
KWB-92	10R	112.0	5R	35.0	R
KWB-93	70S	1137.5	30MS	532.0	S
KWB-94	70S	1120.0	30MS	549.5	S
KWB-95	5R	77.0	0I	0.0	R
KWB-96	70S	1092.0	30MS	502.5	S
KWB-97	40MS	609.0	17R	212.4	MS
KWB-98	10R	185.5	5R	35.0	R
KWB-99	40MS	623.0	20MR	255.5	MS
KWB-100	60S	889.0	20MR	315.0	S
KWB-101	30MR	402.5	10R	140.0	MR
KWB-102	40MS	595.0	10R	147.0	MS
KWB-103	70S	1046.5	30MS	511.0	S
KWB-104	70S	1056.3	30MS	535.5	S
KWB-105	70S	1185.1	40MS	595.0	S
KWB-106	40MS	591.5	10R	197.7	MS
KWB-107	30MR	423.5	10R	154.0	MR
KWB-108	40MS	613.9	16R	204.7	MS
KWB-109	30MR	479.5	13R	147.0	MR
KWB-110	30MR	489.0	25MR	336.0	MR
KWB-111	30MR	445.2	13R	169.7	MR
KWB-112	5R	30.8	5R	18.0	R
KWB-113	5R	30.8	5R	28.0	R

KWB-114	5R	30.8	5R	30.8	R
KWB-115	5R	77.0	0I	0.0	R
KWB-116	60S	868.0	20MR	395.5	S
KWB-117	30MR	416.5	10R	147.0	MR
KWB-118	5R	30.5	5R	14.0	R
KWB-119	10R	105.0	5R	28.0	R
KWB-120	20MR	313.5	10R	128.0	MR
KWB-121	30MR	406.5	10R	228.0	MR
KWB-122	40MS	616.0	10R	190.7	MS
KWB-123	80S	1162.7	40MS	546.0	S
KWB-124	80S	1172.5	40MS	584.5	S
KWB-125	10R	112.0	5R	28.0	R
KWB-126	5R	32.0	0I	0.0	R
KWB-127	80S	1197.0	40MS	532.0	S
KWB-128	40MS	672.0	10R	224.0	MS
KWB-129	5R	35.0	0I	0.0	R
KWB-130	30MR	442.7	10R	134.7	MR
KWB-131	40MS	665.0	10R	210.0	MS
KWB-132	5R	35.0	0I	0.0	R
KWB-133	40MS	630.0	10R	150.5	MS
KWB-134	70S	1107.5	20MR	290.5	S
KWB-135	70S	1064.0	20MR	255.5	S
KWB-136	80S	1249.5	20MR	329.0	S
KWB-137	5R	48.7	5R	21.0	R
KWB-138	5R	60.2	0I	0.0	R
KWB-139	10R	171.5	5R	35.0	R
KWB-140	5R	77.0	0I	0.0	R
KWB-141	70S	1123.5	20MR	357.0	S
KWB-142	70S	1122.4	20MR	346.5	S
KWB-143	5R	35.0	5R	21.0	R
KWB-144	70S	1121.7	20MR	315.0	S
KWB-145	10R	134.0	5R	35.0	R
KWB-146	70S	1120.0	20R	217.0	S
KWB-147	5R	35.0	0I	0.0	R
KWB-148	70S	1092.7	10R	190.7	S
KWB-149	80S	1207.5	10R	169.7	S
KWB-150	50MS	740.6	10R	99.7	MS
KWB-151	40MS	581.0	10R	85.7	MS
KWB-152	40MS	659.1	10R	113.7	MS
KWB-153	40MS	645.4	10R	113.7	MS
KWB-154	70S	1144.5	30MS	502.5	S
KWB-155	80S	1180.9	20R	199.5	S
KWB-156	80S	1200.5	20R	190.7	S
KWB-157	70S	1127.0	20R	232.7	S
KWB-158	30MS	507.5	10R	113.7	MS
KWB-159	50MS	744.1	10R	120.7	MS
KWB-160	30MS	518.0	10R	110.9	MS
KWB-161	50MS	750.1	10R	129.5	MS
KWB-162	40MS	884.1	10R	150.5	MS
KWB-163	50MS	861.0	10R	94.5	MS
KWB-164	75S	1174.6	30MS	508.0	S
KWB-165	70S	1067.5	30MS	540.0	S
KWB- 166	70S	997.5	30MS	526.0	S
KWB- 167	70S	1176.0	30MS	526.0	S
KWB- 168	80S	1218.0	30MS	514.0	S
KWB- 169	80S	1310.4	30MS	537.0	S
KWB- 170	80S	1358.0	30MS	507.0	S
KWB- 171	60S	952.0	30MS	508.0	S
KWB- 172	60S	949.9	30MR	499.0	S
KWB- 173	60S	962.5	30MS	504.0	S

KWB- 174	70S	1098.0	30MS	501.0	S
KWB- 175	70S	978.0	30MS	501.0	S
KWB- 176	60S	749.0	30MR	498.0	S
Avocet-Yr5	5R	21.0	0I	0.0	R
Avocet- Yr10	5R	21.0	0I	0.0	R
Avocet-Yr15	5R	21.0	0I	0.0	R
DBW-187	5R	42.0	0I	0.0	R
KWB- 181	30MR	430.0	10R	110.0	MR
KWB- 182	30MR	417.0	5R	21.0	MR
KWB- 183	20MR	480.0	5R	14.0	MR
KWB- 184	70S	1155.0	33MS	522.0	S
KWB- 185	70S	1060.0	30MS	510.0	S
KWB- 186	40MS	587.0	10R	129.0	MS
Agra-local	90S	1310.0	40MS	670.0	S
PBW-343	80S	1300.0	40MS	636.0	S
Shalimar Wheat-1	70S	1160.0	30MS	520.0	S
Shalimar Wheat-2	60MS	749.0	30MR	410.0	MS
Shalimar Wheat-3	30MR	456.0	10R	190.0	MR
PBW-757	5R	30.8	0I	0.0	R

Abbreviations: **I**= immune, **R**=resistant, **MR**=moderately resistant, **MS**= moderately susceptible, **S**= susceptible .

Table S2. Disease reactions of the wheat genotypes against the six most virulent and aggressive races of *P. striiformis.*

Genotype	7S0	238S119	110S119	110S84	47S119 (T)	46S119
KWB-1	R	S	S	MS	S	MR
KWB-2	R	S	S	MR	R	MR
KWB-3	R	S	S	S	S	S
KWB-5	R	MS	R	R	MR	MR
KWB-6	S	R	S	R	R	MS
KWB-7	R	S	MR	R	R	R
KWB-8	R	R	R	R	R	R
KWB-9	R	MS	S	R	R	MS
KWB-12	R	S	S	S	R	MS
KWB-14	R	S	S	S	R	S
KWB-15	R	S	MS	R	S	MS
KWB-16	R	S	S	S	R	S
KWB-17	R	S	S	MS	R	MR
KWB-19	R	S	S	S	MS	MS
KWB-20	R	S	S	S	S	MR
KWB-21	R	S	S	R	R	R
KWB-22	R	S	S	MS	MS	MS
KWB-23	R	S	S	S	R	S
KWB-24	R	S	S	R	R	S
KWB-25	R	S	MS	R	R	MR
KWB-26	R	S	S	S	MR	MR
KWB-28	R	S	S	R	S	R
KWB-29	R	S	S	R	R	R
KWB-30	R	MS	S	R	R	R
KWB-32	R	MS	R	R	R	MR
KWB- 33	R	S	S	R	S	MS
KWB-34	R	S	S	MS	R	R
KWB-35	R	S	S	MS	MS	MR
KWB-36	R	S	S	S	S	S
KWB- 39	R	S	S	MS	R	R
KWB-41	R	S	S	R	MS	MS
KWB- 42	MR	R	S	MS	MS	S
KWB-43	R	R	R	R	R	R
KWB-44	R	R	MS	S	S	MS
KWB- 45	R	S	S	MS	S	MS
KWB-49	R	S	S	MS	MS	MS
KWB- 50	R	S	S	S	MR	S
KWB- 52	R	S	S	R	R	S
KWB- 53	R	S	S	S	R	MR
KWB- 54	R	MS	R	R	R	R
KWB- 56	R	S	S	R	S	S
KWB- 58	R	MR	MS	MR	MR	R
KWB-59	R	S	S	R	S	MR
KWB- 61	R	S	MS	MR	R	R
KWB-63	R	S	S	R	MS	R
KWB-64	R	R	R	R	R	R
KWB- 65	R	S	S	MR	R	R
KWB- 67	R	MS	S	S	R	S

KWB- 68	MS	S	S	S	R	S
KWB- 69	R	S	MS	MS	R	R
KWB-70	R	S	S	R	S	R
KWB-71	R	S	S	MS	R	S
KWB- 72	R	S	S	MR	MS	R
KWB- 73	R	S	S	R	MR	R
KWB- 74	R	S	MS	MR	R	R
KWB-75	R	S	S	MS	MS	MS
KWB-76	R	S	S	R	R	R
KWB- 77	R	S	R	R	R	R
KWB- 78	R	MS	S	S	R	MS
KWB- 79	R	S	S	R	MS	R
KWB- 80	R	R	S	R	S	R
KWB- 83	R	S	S	R	R	S
KWB- 84	R	S	S	S	S	S
KWB- 85	R	S	S	S	S	MR
KWB- 86	R	S	S	S	R	MR
KWB-87	R	MS	MR	R	MR	R
KWB- 88	R	S	MR	R	MS	R
KWB- 89	R	S	S	S	MS	R
KWB- 90	MS	S	S	S	MS	S
KWB- 91	R	R	R	R	R	R
KWB- 92	MS	S	S	R	MS	R
KWB- 93	R	S	S	R	R	S
KWB-95	R	R	R	R	R	R
KWB- 97	R	S	S	R	MS	S
KWB- 98	R	S	S	R	MS	R
KWB- 101	R	S	S	S	S	MS
KWB-103	R	S	S	MS	R	S
KWB-104	R	S	S	S	R	S
KWB- 105	R	S	S	R	R	MS
KWB- 107	R	S	S	R	S	MR
KWB-108	R	S	S	MS	MR	MS
KWB- 109	R	S	S	MR	MR	MR
KWB- 112	R	S	S	R	R	R
KWB- 113	MS	S	MR	R	R	R
KWB-114	R	S	S	R	R	R
KWB-115	R	R	R	R	R	R
KWB- 116	R	S	S	MS	R	MS
KWB- 117	R	S	S	R	MR	MR
KWB-118	R	S	R	R	R	R
KWB-119	R	S	S	S	R	R
KWB- 120	R	MS	MR	R	R	MR
KWB- 121	R	S	S	S	S	MS
KWB-123	R	S	S	S	R	S
KWB-125	R	MS	R	R	R	R
KWB- 126	R	R	R	R	R	R
KWB- 129	R	R	R	R	R	R
KWB- 132	R	R	R	R	R	R
KWB- 136	R	S	S	S	MS	S
KWB- 138	R	R	R	R	MR	R

KWB- 140	R	R	R	MR	R	MR
KWB- 142	R	S	S	S	S	S
KWB- 143	R	S	R	S	R	MS
KWB-144	R	S	R	S	R	S
KWB- 147	R	R	R	R	R	R
KWB-148	R	S	S	S	S	S
KWB-149	R	S	S	S	S	S
KWB-163	S	S	S	S	S	S
SW1	R	S	S	S	R	S
SW2	R	R	S	S	MS	MS
SW3	R	R	S	R	R	MR

R = resistant, S = susceptible, MR = moderately resistant, MS = moderately susceptible.

Table S3. Molecular screening of wheat genotypes for presence of four effective yellow rust resistance genes.

Genotype	*Yr5* (STS7/8)	*Yr10* (XPSP3000)	*Yr15* (Xbarc8)	*Yr17* (VENTRUIP/LN-2)
KWB-1	-	-	-	-
KWB-2	-	-	-	+
KWB-4	-	-	-	-
KWB-5	+/-	-	-	-
KWB-6	-	-	-	-
KWB-7	-	-	-	+
KWB-8	-	+	+	+
KWB-16	-	-	-	-
KWB-17	-	-	-	-
KWB-18	-	-	-	-
KWB-20	-	-	-	-
KWB-21	-	-	+/-	-
KWB-22	-	-	-	-
KWB-23	-	-	-	-
KWB-27	-	-	-	-
KWB-28	-	-	-	+
KWB-30	-	-	-	+
KWB-32	-	-	-	-
KWB-33	-	-	-	-
KWB-35	-	-	-	-
KWB-37	-	-	-	+
KWB-39	-	-	-	+
KWB-40	-	+/-	-	+
KWB-41	-	-	-	-
KWB-43	+	+	-	+
KWB-44	-	-	-	-
KWB-46	-	-	-	-
KWB-50	-	-	-	-
KWB-53	-	-	-	-
KWB-54	-	-	-	-
KWB-55	-	-	-	-
KWB-56	-	-	-	-
KWB-58	-	-	-	+
KWB-59	-	-	-	-
KWB-61	-	-	-	+
KWB-63	-	-	-	-
KWB-64	-	+	+	+
KWB-65	-	-	-	-
KWB-68	-	-	-	-
KWB-72	-	-	-	+
KWB-73	-	-	-	-
KWB-74	-	-	-	+
KWB-76	-	-	-	+
KWB-77	-	-	-	-

KWB-78	-	-	-	-
KWB-79	-	-	-	+
KWB-80	-	-	-	+
KWB-82	-	-	-	-
KWB-85	-	-	-	-
KWB-86	-	-	-	-
KWB-87	-	-	-	+
KWB-89	-	-	-	-
KWB-91	-	+/-	+	+
KWB-95	+	+	-	+
KWB-98	-	-	-	+
KWB-100	-	-	-	-
KWB-102	-	-	-	-
KWB-103	-	-	-	-
KWB-105	-	-	-	-
KWB-108	-	-	-	-
KWB-111	-	-	-	-
KWB-112	-	-	-	+
KWB-113	-	-	-	+
KWB- 115	-	+/-	+/-	+
KWB-119	-	-	-	+
KWB-120	-	-	-	-
KWB-121	-	-	-	-
KWB-125	-	-	-	-
KWB- 126	-	+/-	+	+
KWB-128	-	-	-	-
KWB-129	+/-	-	+	+
KWB-132	+	+	-	+
KWB-135	-	-	-	-
KWB-137	+/-	-	+/-	-
KWB-138	-	+/-	+/-	+
KWB-140	+/-	-	+/-	+
KWB-141	-	-	-	-
KWB-143	-	-	-	-
KWB- 146	-	-	-	-
KWB-147	+	-	+/-	+
KWB-148	-	-	-	-
KWB-149	-	-	-	-
KWB-167	-	-	-	-
KWB-168	-	-	-	-
KWB-184	-	-	-	-
KWB- 186	-	-	-	-
Shalimar Wheat-1	-	-	-	-
Shalimar Wheat-2	-	-	-	-
Shalimar Wheat-3	-	-	-	-
Agra-local	-	-	-	-
Avocet Yr5	+	-	-	-
Avocet Yr10	-	+	-	-
Avocet Yr15	-	-	+	-

Unnat PBW-343	-	-	-	+
PBW-703	-	+	+	-
PBW-757	-	-	+	-

Table S4. Details of genotypes used in the present study.

S. No	Name of genotype	Source / Pedigree	Origin
1.	KWB-1	9thHPAN- -1	Exotic
2.	KWB-2	9thHPAN- CIMMYT-2	Exotic
3.	KWB-3	9thHPAN-CIMMYT-3	Exotic
4.	KWB-4	9thHPAN-CIMMYT-4	Exotic
5.	KWB-5	9thHPAN- CIMMYT-5	Exotic
6.	KWB-6	9thHPAN- CIMMYT-6	Exotic
7.	KWB-7	9thHPAN- CIMMYT-7	Exotic
8.	KWB-8	9thHPAN- CIMMYT-8	Exotic
9.	KWB-9	9thHPAN- CIMMYT-9	Exotic
10.	KWB-10	9thHPAN- CIMMYT-10	Exotic
11.	KWB-11	9thHPAN- CIMMYT-11	Exotic
12.	KWB-12	9thHPAN-CIMMYT-12	Exotic
13.	KWB-13	9thHPAN-CIMMYT-13	Exotic
14.	KWB-14	9thHPAN- CIMMYT-14	Exotic
15.	KWB-15	9thHPAN- CIMMYT-15	Exotic
16.	KWB-16	9thHPAN- CIMMYT-16	Exotic
17.	KWB-17	9th HPAN- CIMMYT-17	Exotic
18.	KWB-18	9th HPAN- CIMMYT-18	Exotic
19.	KWB-19	9thHPAN-CIMMYT-19	Exotic
20.	KWB-20	9thHPAN- CIMMYT-20	Exotic
21.	KWB-21	9thHPAN- CIMMYT-21	Exotic
22.	KWB-22	9thHPAN- CIMMYT-22	Exotic
23.	KWB-23	9th HPAN- CIMMYT-23	Exotic
24.	KWB-24	9th HPAN- CIMMYT-24	Exotic
25.	KWB-25	9th HPAN- CIMMYT-25	Exotic
26.	KWB-26	9th HPAN- CIMMYT-26	Exotic
27.	KWB-27	9th HPAN- CIMMYT-27	Exotic
28.	KWB-28	9th HPAN- CIMMYT-28	Exotic
29.	KWB-29	9th HPAN- CIMMYT-29	Exotic
30.	KWB-30	9th HPAN- CIMMYT-30	Exotic
31.	KWB-31	9th HPAN- CIMMYT-31	Exotic
32.	KWB-32	9th HPAN- CIMMYT-32	Exotic
33.	KWB- 33	9th HPAN- CIMMYT-33	Exotic
34.	KWB-34	9th HPAN- CIMMYT-34	Exotic
35.	KWB-35	9th HPAN- CIMMYT-35	Exotic
36.	KWB-36	9th HPAN- CIMMYT-36	Exotic
37.	KWB-37	9th HPAN- CIMMYT-37	Exotic
38.	KWB-38	9th HPAN- CIMMYT-38	Exotic
39.	KWB- 39	9th HPAN- CIMMYT-39	Exotic
40.	KWB-40	9th HPAN- CIMMYT-40	Exotic
41.	KWB-41	9th HPAN- CIMMYT-41	Exotic
42.	KWB- 42	9th HPAN- CIMMYT-42	Exotic
43.	KWB-43	9th HPAN- CIMMYT-43	Exotic
44.	KWB-44	9th HPAN- CIMMYT-44	Exotic
45.	KWB- 45	9th HPAN- CIMMYT-45	Exotic
46.	KWB- 46	26th SAWYT-376	Exotic
47.	KWB- 47	HRWSN-2085	Exotic
48.	KWB- 48	EIGN 39A	Exotic
49.	KWB-49	IBWSN-1153	Exotic

50.	KWB- 50	EIGN-4A	Exotic
51.	KWB-51	9th CIMMYT-HPAN-51	Exotic
52.	KWB- 52	9th CIMMYT-HPAN-52	Exotic
53.	KWB- 53	9th CIMMYT-HPAN-53	Exotic
54.	KWB- 54	9th CIMMYT-HPAN-54	Exotic
55.	KWB- 55	9th CIMMYT-HPAN-55	Exotic
56.	KWB- 56	SAWSN-3008	Exotic
57.	KWB-57	SAWSN-3089	Exotic
58.	KWB- 58	19th DSBW-O183	Exotic
59.	KWB-59	ESWYT-146	Exotic
60.	KWB-60	MLT-W 2103	Indigenous
61.	KWB- 61	SAWSN-3029	Exotic
62.	KWB-62	SAWSN-3028	Exotic
63.	KWB-63	36-SAWSN-V3098	Exotic
64.	KWB-64	36-SAWSN-3105	Exotic
65.	KWB- 65	36-SAWSN-3172	Exotic
66.	KWB-66	36-SAWSN-3201	Exotic
67.	KWB- 67	36-SAWSN-3182	Exotic
68.	KWB- 68	36-SAWSN-3183	Exotic
69.	KWB- 69	36-SAWSN-3226	Exotic
70.	KWB-70	36-SAWSN-3292	Exotic
71.	KWB-71	36-SAWSN-V3062	Exotic
72.	KWB- 72	26-HRWYT-V213	Exotic
73.	KWB- 73	26-HRWYT-V237	Exotic
74.	KWB- 74	26-HRWYT-V243	Exotic
75.	KWB-75	26-HRWYT-V234	Exotic
76.	KWB-76	26-HRWYT-V209	Exotic
77.	KWB- 77	26-HRWYT-V233	Exotic
78.	KWB- 78	26-HRWYT-V206	Exotic
79.	KWB- 79	7-WYCYT-CIMMYT-V10	Exotic
80.	KWB- 80	7-WYCYT-CIMMYT-V11	Exotic
81.	KWB-81	7-WYCYT-CIMMYT-03	Exotic
82.	KWB-82	7-WYCYT-CIMMYT-28	Exotic
83.	KWB- 83	7-WYCYT-CIMMYT-V21	Exotic
84.	KWB- 84	7-WYCYT-CIMMYT-V18	Exotic
85.	KWB- 85	7-WYCYT-CIMMYT-V8	Exotic
86.	KWB- 86	7-WYCYT-CIMMYT-V31	Exotic
87.	KWB-87	6-WYCYT-CIMMYT-V18	Exotic
88.	KWB- 88	6-WYCYT-CIMMYT-V13	Exotic
89.	KWB- 89	6-WYCYT-CIMMYT-V7	Exotic
90.	KWB- 90	36-SAWSN-V3099	Exotic
91.	KWB- 91	36-SAWSN-V3116	Exotic
92.	KWB- 92	36-SAWSN-V3293	Exotic
93.	KWB- 93	36-SAWSN-V3255	Exotic
94.	KWB-94	36-SAWSN-V3076	Exotic
95.	KWB-95	36-SAWSN-V3234	Exotic
96.	KWB-96	36-SAWSN-V3077	Exotic
97.	KWB- 97	36-SAWSN-V3048	Exotic
98.	KWB- 98	36-SAWSN-V3199	Exotic
99.	KWB-99	36-SAWSN-V3268	Exotic
100.	KWB-100	36-SAWSN-V3071	Exotic
101.	KWB- 101	36-SAWSN-V3233	Exotic
102.	KWB-102	36-SAWSN-V3265	Exotic
103.	KWB-103	36-SAWSN-V3213	Exotic
104.	KWB-104	36-SAWSN-V3212	Exotic
105.	KWB- 105	36-SAWSN-V3088	Exotic
106.	KWB-106	39-ESWYT-CIMMYT-V111	Exotic

107.	KWB- 107	39-ESWYT-CIMMYT-V132	Exotic
108.	KWB-108	39-ESWYT-CIMMYT-V144	Exotic
109.	KWB- 109	39-ESWYT-CIMMYT-V113	Exotic
110.	KWB- 110	40-ESWYT-CIMMYT-V122	Exotic
111.	KWB- 111	40-ESWYT-CIMMYT-V141	Exotic
112.	KWB- 112	40-ESWYT-CIMMYT-V105	Exotic
113.	KWB- 113	40-ESWYT-CIMMYT-V102	Exotic
114.	KWB-114	29th HRWSN 2065	Exotic
115.	KWB-115	9th HPAN-CIMMYT-12	Exotic
116.	KWB- 116	9th HPAN-CIMMYT-87	Exotic
117.	KWB- 117	ESWYT-120-G-101	Exotic
118.	KWB-118	HPAN-CIMMYT-G-36	Exotic
119.	KWB-119	HPAN-CIMMYT-10-G-10	Exotic
120.	KWB- 120	HPAN-CIMMYT-8-G-8	Exotic
121.	KWB- 121	DSWYT-29406-G-106	Exotic
122.	KWB-122	NHIVT-1813-G-7	Indigenous
123.	KWB-123	9th HPAN-CIMMYT-G-58	Exotic
124.	KWB-124	VW-1807-G-37	Indigenous
125.	KWB-125	VW-1807-G-28	Indigenous
126.	KWB- 126	SBWYT-26-G-85	Exotic
127.	KWB- 127	NHIVT-1807-G-2	Indigenous
128.	KWB- 128	HRWYT-53- G-79	Exotic
129.	KWB- 129	NHIVT-1801-G-1	Indigenous
130.	KWB- 130	VW-1812-G66	Indigenous
131.	KWB- 131	Wheat-12-G-56	Exotic
132.	KWB- 132	HPAN-CIMMYT-90- G32	Exotic
133.	KWB- 133	SAWSN-3081-G47	Exotic
134.	KWB- 134	SBW4727-G86	Exotic
135.	KWB- 135	HPAN-CIMMYT-9-G9	Exotic
136.	KWB- 136	HPAN-CIMMYT-6-G6	Exotic
137.	KWB- 137	HPAN-CIMMYT-100-G43	Exotic
138.	KWB- 138	HLBSN-34	Exotic
139.	KWB-139	HPAN-CIMMYT-11-G12	Exotic
140.	KWB- 140	HPAN-CIMMYT-84	Exotic
141.	KWB-141	HLBSM-32-G71	Exotic
142.	KWB- 142	NHIVT-1805-G5	Indigenous
143.	KWB- 143	NHIVT-1805-G6	Indigenous
144.	KWB-144	HPAN-CIMMYT-90- G32	Exotic
145.	KWB-145	HPAN-CIMMYT- 19-G-20	Exotic
146.	KWB-146	VW-1810-G59	Indigenous
147.	KWB- 147	HRWYT-84-G80	Exotic
148.	KWB-148	HTWYT-64-G87	Exotic
149.	KWB-149	HPAN-CIMMYT- 88-G30	Exotic
150.	KWB-150	HRWSN-2066-G74	Exotic
151.	KWB-151	SAWSN-3080-G65	Exotic
152.	KWB-152	HPAN-CIMMYT- 20-G21	Exotic
153.	KWB-153	HPAN-CIMMYT- 89-G31	Exotic
154.	KWB-154	HPAN-CIMMYT- 96-G70	Exotic
155.	KWB-155	HLBSN-31-G39	Exotic
156.	KWB-156	VW-1811-G63	Indigenous
157.	KWB-157	SAWSN-3079-G69	Exotic
158.	KWB-158	HPAN-CIMMYT- 96-G39	Exotic
159.	KWB-159	HPAN-CIMMYT- 92-G35	Exotic
160.	KWB-160	HPAN-CIMMYT- 91-G34	Exotic
161.	KWB-161	HPAN-CIMMYT- 85-G27	Exotic
162.	KWB-162	NHIVT-1803-G3	Indigenous
163.	KWB-163	NHIVT-1614	Indigenous

164.	KWB-164	19th DSBW-ON-199-G98	Exotic
165.	KWB-165	Ladakh Local 1	Indigenous
166.	KWB-166	Ladakh Local 2	Indigenous
167.	KWB-167	Ladakh Local 3	Indigenous
168.	KWB-168	Ladakh Local 4	Indigenous
169.	KWB-169	Ladakh Local 5	Indigenous
170.	KWB-170	Ladakh Local 6	Indigenous
171.	KWB-171	Ladakh Local 7	Indigenous
172.	KWB-172	Ladakh Local 8	Indigenous
173.	KWB-173	Ladakh Local 9	Indigenous
174.	KWB-174	Ladakh Local 10	Indigenous
175.	KWB-175	Ladakh Local 11	Indigenous
176.	KWB-176	Ladakh Local 12	Indigenous
177.	Avocet Yr5	Avocet Yr5	Exotic
178.	Avocet Yr10	Avocet Yr10	Exotic
179.	Avocet Yr15	Avocet Yr15	Exotic
180.	PBW757	PBW757	Indigenous
181.	DBW-187	DBW187	Indigenous
182.	KWB-181	Ladakh Local 16	Indigenous
183.	KWB-182	SKW356	Indigenous
184.	KWB-183	8th SATYN 9408	Exotic
185.	KWB-184	36th SAWSN 3081	Exotic
186.	KWB-185	8th SATYN 9431	Exotic
187.	KWB-186	HTWYT-7	Exotic
188.	Agra-Local	Agra-Local	Indigenous
189.	PBW-343	PBW-343	Indigenous
190.	SW1	SW1	Indigenous
191.	SW2	SW2	Indigenous
192.	SW3	SW3	Indigenous

Table S5. Disease reaction type and Phytopathological symptoms observations.

Disease reaction type	Phytopathological symptoms observed
Immune	0; (naught fleck- no visible infection); (fleck-no uredia but small hypersensitivity flecks present); (micro-flecking-very light necrosis)
R (Resistant)	1 (Necrotic areas with or without minute uredia)
MR (Moderately Resistant)	2,2- (Small uredia present surrounded by necrotic area)
MRMS (Moderately Resistant-Moderately Susceptible)	2+,22+,3- (Small uredia present surrounded bynecrotic areas as well as medium uredia with no necrosis with some distinct chlorosis)
MS (Moderately Susceptible)	3,3- (Medium uredia with no necrosis with some distinct chlorosis)
MSS (Moderately Susceptible-Susceptible)	33+ (Medium uredia with no necrosis but possible some distinct chlorosis as well as large uredia with little orchlorosis present)
S(Susceptible)	3+ (Large uredia and little or no chlorosis present)
